# The Feasibility of a Telehealth Exercise Program Aimed at Increasing Cardiorespiratory Fitness for People After Stroke

**DOI:** 10.5195/ijt.2019.6290

**Published:** 2019-12-12

**Authors:** Margaret Galloway, Dianne L. Marsden, Robin Callister, Michael Nilsson, Kirk I. Erickson, Coralie English

**Affiliations:** 1 School of Health Sciences, Faculty of Health and Medicine, University of Newcastle, Callaghan, NSW, Australia; 2 Hunter Stroke Service, Hunter New England Local Health District, Newcastle, NSW, Australia; 3 School of Biomedical Sciences and Pharmacy, Faculty of Health and Medicine, University of Newcastle, Callaghan, NSW, Australia; 4 School of Medicine and Public Health, Faculty of Health and Medicine, University of Newcastle, Callaghan, NSW, Australia; 5 Centre for Rehab Innovations, Hunter Medical Research Institute, New Lambton, NSW, Australia; 6 Department of Psychology, University of Pittsburgh, Pittsburgh, Pennsylvania, USA

**Keywords:** Cardiorespiratory fitness, Exercise, Stroke, Telemedicine, Telerehabilitation

## Abstract

**Background:**

Accessing suitable fitness programs post-stroke is difficult for many. The feasibility of telehealth delivery has not been previously reported.

**Objectives:**

To assess the feasibility of, and level of satisfaction with home-based telehealth-supervised aerobic exercise training post-stroke.

**Methods:**

Twenty-one ambulant participants (≥ 3 months post-stroke) participated in a home-based telehealth-supervised aerobic exercise program (3 d/week, moderate-vigorous intensity, 8-weeks) and provided feedback via questionnaire post-intervention. Session details, technical issues, and adverse events were also recorded.

**Results:**

Feasibility was high (83% of volunteers met telehealth eligibility criteria, 85% of sessions were conducted by telehealth, and 95% of participants rated usability favourably). Ninety-five percent enjoyed telehealth exercise sessions and would recommend them to others. The preferred telehealth exercise program parameters were: frequency 3 d/week, duration 20-30 min/session, program length 6-12 weeks.

**Conclusion:**

The telehealth delivery of exercise sessions to people after stroke appears feasible and may be considered as a viable alternative delivery means for providing supervised exercise post-stroke.

Many people after stroke are not meeting the minimum recommendations included in physical activity guidelines (20 - 30 minutes of moderate exercise on most days) (Billinger et al., 2014; English, Manns, Tucak, & Bernhardt, 2014; Fini, Holland, Keating, Simek, & Bernhardt, 2017; Royal College of Physicians, 2016; Stroke Foundation, 2017; Thilarajah et al., 2018). This can increase both the risk and the severity of secondary stroke (Barengo, Antikainen, Borodulin, Harald, & Jousilahti, 2017; O'Donnell et al., 2016; Reinholdsson, Palstam, & Sunnerhagen, 2018; Saunders, Greig, & Mead, 2014) and increase the risk of all-cause mortality (Lee et al., 2011). In addition, low physical activity may also contribute to a lower quality of life after stroke as activities of daily living become more difficult due to further decline in mobility and cardiorespiratory fitness through inactivity (Billinger, Coughenour, Mackay-Lyons, & Ivey, 2012). Increasing physical activity post-stroke may also improve cognitive function (Cumming, Tyedin, Churilov, Morris, & Bernhardt, 2012), walk speed, functional mobility, muscle strength, bone density and quality of life (Morris, Macgillivray, & McFarlane, 2014). Providing ways for people who have experienced a stroke to access suitable exercise programs is critical in addressing the very low cardiorespiratory fitness levels seen in this population (Marsden, Dunn, Callister, Levi, & Spratt, 2013; Saunders et al., 2016; Smith, Saunders, & Mead, 2012). Studies included in these reviews have reported significant gains in cardiorespiratory fitness with a range of exercise interventions post-stroke, lowering secondary stroke risk and all-cause mortality (Keteyian et al., 2008). Cardiorespiratory fitness gains and adherence to exercise are often higher if exercise programs are supervised (Hwang, Bruning, Morris, Mandrusiak, & Russell, 2015; Olney et al., 2006), however a number of barriers exist that make participating in centre–based exercise programs difficult. These include (i) logistical factors (such as location, family support and access to transport), (ii) psycho-social factors (motivation, confidence, and exercise knowledge), and (iii) factors more directly related to the consequences of the stroke, such as stroke-related physical or cognitive impairments (Jackson, Mercer, & Singer, 2018; Nicholson et al., 2013; Prout, Mansfield, McIlroy, & Brooks, 2017; Tyagi et al., 2018). Delivering exercise programs via telehealth using video-conferencing may provide an alternative way to enable stroke survivors to engage in supervised exercise at home.

Telehealth exercise interventions targeting fitness have proved effective and safe in other populations, including cardiac rehabilitation (Clark et al., 2015), chronic obstructive pulmonary disease (Hwang et al., 2015), cystic fibrosis (Cox, Alison, Button, Wilson, & Holland, 2015) and in the elderly (Crotty et al., 2014), and telehealth is emerging as a cost-effective alternative to providing centre-based exercise programs (Maddison et al., 2018; Southard, Southard, & Nuckolls, 2003; Whittaker & Wade, 2014). In stroke, telehealth has been used successfully to monitor vital signs (Bernocchi et al., 2016), motor recovery, depression and higher cortical function (Sarfo, Ulasavets, Opare-Sem, & Ovbiagele, 2018), and to deliver speech pathology (Hill et al., 2006), upper limb physiotherapy (Benvenuti et al., 2014) and rehabilitation exercises (Bernocchi et al., 2016). However, a recent systematic review of telerehabilitation interventions for stroke recovery did not find any trials that assessed the feasibility of delivering exercise programs aimed specifically at increasing cardiorespiratory fitness (Sarfo et al., 2018), and little is known about the safety or user experience for this population.

We do not know whether there are barriers for people after stroke to access or engage in telehealth supervised exercise programs. It is possible that stroke-related physical or cognitive impairments may have an impact on feasibility. Additionally, age or technology-related barriers (such as access to suitable internet speed or devices, or prior familiarity with technology) may prevent or discourage some stroke survivors from engaging in exercise programs via telehealth. In other clinical populations telehealth delivery of exercise has been effective in reducing one of the main barriers to exercise (transport) (Clark et al., 2015; Evenson & Fleury, 2000; Ragupathi et al., 2017). As few studies have looked at the feasibility of the telehealth delivery of fitness program after stroke, there is also little information available about the acceptability of telehealth exercise programs in terms of preferred dosage (session frequency, duration, program length, or exercise mode) or likely future use.

In a recent study, we determined the tolerability of low doses of exercise and their effect on cardiorespiratory fitness in community-dwelling ambulant stroke survivors (Galloway, 2019). The exercise intervention in that trial was focused on increasing cardiorespiratory fitness and was delivered primarily via telehealth. The purpose of the current study was to determine the feasibility and acceptability of telehealth-supervised aerobic exercise from the perspective of both the participant and the research team. The specific research questions for the current study were:

- How feasible is it to deliver supervised exercise by telehealth; specifically,
  - Does the telehealth delivery influence recruitment, retention, and adherence?
  - How safe were exercise sessions delivered by telehealth?
  - How reliable was the telehealth platform in terms of connectivity and video/audio quality?
  - How usable was the telehealth platform for participants?
- How satisfied were participants with:
  - Telehealth delivery of a home-based aerobic exercise program, and did prior level of technical familiarity, age or level of disability affect satisfaction?
  - The dose of exercise delivered?
  - The content of exercise sessions?

## METHODS

### STUDY DESIGN

This study evaluated the feasibility and level of participant satisfaction of an 8-week, home-based, telehealth-supervised aerobic exercise program for people after stroke, and was part of a larger study (Galloway, 2019). Feasibility was assessed by recruitment, retention, program adherence, adverse events and the reliability and quality of telehealth delivery. Information regarding participation, safety and technical issues were recorded by the exercise instructor at the completion of each exercise session. The level of satisfaction with telehealth delivery and the exercise program were evaluated using participant feedback via a questionnaire and follow up phone call within 4 weeks of completing the exercise program.

### ETHICS AND REGISTRATION

This study was approved by the Hunter New England Human Research Ethics Committee (HNEHREC Reference No: 16/10/19/4.09) and also registered with the University of Newcastle Human Research Ethics Committee (H-2017-0045). All participants were provided with written information about the study and gave written informed consent. The larger study was registered with the Australian New Zealand Clinical Trials Registry.

### RECRUITMENT AND ELIGIBILITY CRITERIA OF PARTICIPANTS

Participants were recruited via the Hunter Stroke Research Register, social media, databases of previous study participants who had agreed to be contacted for future studies, and by word of mouth. Individuals were eligible if they were: community-dwelling, ≥ 18 y, ≥ 3 months post-stroke, had clearance from a medical practitioner to participate, and were able to walk independently (score ≥ 3 on the Functional Ambulation Classification [FAC])(Holden, Gill, Magliozzi, Nathan, & Piehl-Baker, 1984). Participants were also required to have internet access (broadband, preferably not via a mobile or cellular network), a device such as a laptop, desktop computer or android tablet with a Google Chrome browser or an iPad (also available for loan if required), access to a suitable exercise space, and were able to ensure a responsible person was available at home in case of emergencies during exercise sessions. Individuals were ineligible if they were unable to understand instructions in English, pregnant or planning to be pregnant during the study period, unable to understand two simple commands, had a self-reported current physical activity level greater than moderate intensity for more than 20 min 3 d/week, had a clinical diagnosis of an acute/chronic illness or any other conditions with known exercise contraindications or which limited their ability to complete the fitness assessments or intervention, were unable to commit to all study requirements, or were currently participating in either a stroke research trial or rehabilitation therapy focused on encouraging participation in physical activity.

### EXERCISE INTERVENTION AND DELIVERY

Participants underwent an 8-week, 3d/week home-based, individually-prescribed, aerobic exercise program at moderate to vigorous intensity (Norton, Norton, & Sadgrove, 2010) (55-85% of maximum heart rate as determined at the baseline fitness assessment, or at a Borg rating of perceived exertion [RPE] between 13 and 16) (Borg, 1982). Participants were enrolled sequentially in 4 cohorts (n=5/cohort) therefore the target total sample size was n=20. Session duration varied by cohort (Cohort 1 = 10 min/session, Cohort 2 = 15 min; Cohort 3 = 20 min, Cohort 4 = 20 min) for the entire 8–week program. We planned to supervise all exercise sessions by telehealth (video-conference). If mutually convenient times were not found, participants were encouraged to complete sessions unsupervised. Telehealth video-conference sessions were delivered using proprietary software, via a website (www.neorehab.com) or app (eHab®). If video-conferencing was not available due to technical issues, sessions were to be conducted by phone. All participants were provided with a home visit by a member of the research team prior to the commencement of the intervention where a risk assessment of the identified exercise space was conducted. At this visit participants were assessed on their ability to safely perform the exercises planned for their intervention and taught how to perform all exercises safely. If participants were unable to perform the proposed exercises, these exercises were modified or alternative exercises identified. Participants and/or their responsible person were taught how to access the video-conferencing software and how to monitor post-exercise heart rates using either a pulse oximeter (Crucial Medical Solutions Model CMS50DL) or a heart rate monitor incorporating a chest strap and wrist watch (Garmin Forerunner 25). A safety (emergency) plan was also discussed with the participant and their responsible person.

All telehealth exercise sessions were conducted by one member of the research team (MG, exercise scientist, trained by author DM, a physiotherapist experienced in delivering similar post-stroke exercise programs in-person). Full details of the intervention described according to the template for intervention description and replication (TIDieR) checklist (Hoffmann et al., 2014) are published elsewhere (Galloway, 2019). Briefly, exercise sessions consisted of a number of 5-min blocks of exercise, with higher and lower intensity intervals within each 5-min block. Exercise programs were structured to include a variety of whole, lower and upper body aerobic exercises to minimise local muscular fatigue during sessions. Exercise selection was modelled on a previous study (Marsden et al., 2016), and adapted according to participants' ability. Typical exercises included repetitions of sit to stand exercises, squats, marching on the spot, steps ups using an aerobic step (supplied) and a range of other upper and lower body exercises aimed at increasing heart rate. All programs followed a generic design and were progressive in nature, and adapted individually to take into account initial fitness level, degree of disability, and participant exercise preference. Clinical judgement was used to modify exercises if target heart rates were not achieved or participants experienced any injury, overuse, or discomfort. A questionnaire was administered weekly asking participants whether they had experienced any illnesses, injuries, or falls, and whether they were able to do all their normal activities in the preceding week. Cardiorespiratory fitness (VO_2peak_) was measured by indirect spirometry at Week 0 and Week 8 during a 6-minute walk test and a cycle ergometer graded exercise test.

### PARTICIPANT CHARACTERISTICS

Demographic details were obtained during the baseline assessment and/or at screening. Participants' level of technical familiarity was assessed via questionnaire (Supplementary material). Participants were asked the mode, duration, intensity, and frequency of any regular physical activity they did including walking during screening.

### FEASIBILITY AND ACCEPTABILITY MEASURES

#### RECRUITMENT AND RETENTION:

The number of participants who were screened, eligible, enrolled and completed the study were recorded. The main sources of successful recruitment were identified. Reasons participants were not eligible or did not complete the study were also recorded.

#### PROGRAM ADHERENCE:

The number of both supervised and unsupervised sessions were recorded by the exercise instructor.

#### SAFETY:

The type, circumstances and consequences of any adverse events were recorded.

#### RELIABILITY, QUALITY AND USABILITY OF TELEHEALTH DELIVERY:

Internet reliability, video and audio quality from the perspective of the exercise instructor were recorded each session. Audio and video quality and system usability from the participants' perspective were assessed via questionnaire at the completion of the trial.

#### SATISFACTION:

Participant satisfaction with the telehealth delivery of exercise sessions, the content of the exercise sessions, and participants' preferred dose were assessed via questionnaire at the completion of the trial.

### DATA COLLECTION

Data recorded each exercise session: (i) session details (participant attendance, wellness as self-reported in response to a standard question on illnesses, soreness or injuries that may prevent the participant from exercising that day), adherence to exercise dose, exercise intensities, RPEs, training heart rates, adverse events, and (ii) telehealth related factors (ratings of video quality, audio quality and internet reliability, and reasons for missed or interrupted sessions). Video and audio quality were rated on a 3-point Likert Scale (fair, acceptable or excellent) by the exercise instructor, who also noted whether exercises were being done correctly, and whether participants were experiencing any distress or difficulty.

#### PARTICIPANT FEEDBACK:

Two questionnaires were mailed to participants after their final fitness assessment following the conclusion of their 8-week program. Participants were asked to complete the questionnaires in their own time with help from family members or carers if required. If someone other than the participant was responsible for managing the technology during the intervention, we asked that they provide feedback on those aspects. An independent person phoned participants 1-2 weeks later to collect responses.

#### TECHNICAL FAMILIARITY QUESTIONNAIRE:

To determine whether prior familiarity with technology affected the telehealth experience, participants were asked about their level of engagement with computers, the internet and mobile phones using 15 multiple choice questions adapted from a previously validated questionnaire (O'Brien et al., 2015). Items from this questionnaire were modified slightly to reflect recent changes in technology (e.g., type of internet connection and mobile phone functions). We scored responses across 3 domains (computer usage, internet usage and mobile phone usage), and weighted the responses from each domain evenly to produce a technical familiarity score out of 100.

#### TELEHEALTH USABILITY AND SATISFACTION QUESTIONNAIRE:

The feedback questionnaire included 23 multiple choice questions and 2 open-ended questions. The multiple choice questions used a 5-point Likert-scale, and some items from a previously validated questionnaire were included (Parmanto, Lewis, Graham, & Bertolet, 2016). This questionnaire (the Telehealth Usability Questionnaire) measures the quality of the user interface and the telehealth interaction and services. We also asked participants about their satisfaction with the telehealth delivery and content of the exercise program.

Two experienced stroke researchers independently assessed the face validity of the modified questionnaires during the development phase and changes were made based on their recommendations. Both questionnaires were piloted by two people with stroke during the development stage for readability and use of language (aphasia friendly/plain English).

### STATISTICAL ANALYSIS

Quantitative data were analysed using descriptive statistics. Data from the two open-ended questions in the feedback questionnaire were extracted manually and analysed thematically.

## RESULTS

### RECRUITMENT, RETENTION AND PARTICIPANT CHARACTERISTICS

Sixty-six people who expressed an interest in participating in the telehealth exercise study were screened for eligibility. Forty-two people declined or were ineligible. Thirteen declined for reasons related to telehealth: nine were unable to nominate an adult who was able to be present during planned exercise sessions, two did not have suitable internet access (no broadband connection, or mobile phone connectivity deemed inadequate at their location), and two declined as they were unwilling or unable to operate telehealth systems or had concerns about their ability to do so (Table 1). The main source of recruitment was the Hunter Stroke Research Register (n=13, 62%). Other sources were: social and traditional media, databases of previous study participants who had agreed to be contacted for future studies, and word of mouth.

**Table 1.** Recruitment, Supervision and Adherence of Participants to Telehealth, and the Reliability of Telehealth Delivery

Characteristic	
Recruitment, n recruited/n screened	24/66
Withdrawals, n	3
Completed, n	21
Ineligible to receive telehealth exercise delivery	
No suitable person at home, n (%)	9 (14)
No suitable internet access, n (%)	2 (3)
No suitable device, n (%)	0 (0)
Declined telehealth delivery	
Unable/unwilling to operate device, n (%)	1 (2)
Concerned about ability to manage telehealth (IT), n (%)	1 (2)
Home internet access (n=21)	
High speed broadband (NBN), number (%)	13 (62)
ADSL broadband, number (%)	8 (38)
Devices used (n=20)	
iPad, n (%)	12 (60)
Laptop, n (%)	8 (40)
Android Tablet, n (%)	0 (0)
Sessions completed, n (% of scheduled)	476 (94)
Sessions delivered by telehealth, n (% of scheduled telehealth sessions)	408 (85)
By video-conference, n (% of scheduled telehealth sessions)	372 (78)
By phone, n (% of scheduled telehealth sessions)	20 (4)
By phone and video-conference, n (% of scheduled telehealth sessions)	16 (3)
Reasons telehealth sessions were not delivered by video-conference	
Internet issues, n sessions (%)	24 (5)
Computer issues, n sessions (%)	8 (2)
Video-conference software issues, n sessions, (%)	4 (1)
Other, n sessions (%)	2 (0.4)
Sessions delivered face to face, n (% of scheduled sessions)	14 (3)
Sessions completed unsupervised, n (% of scheduled sessions)	54 (11)
Missed Sessions, n (% of scheduled)	28 (6)
Reasons for missed sessions, n (% of scheduled)	
Illness	16 (3)
Minor Injury/soreness	3 (0.6)
Participant forgot	2 (0.4)
Scheduling conflicts	5 (1)
Public Holiday	1 (0.2)
Technical issues	1 (0.2)

Twenty-four participants were enrolled and n=21 completed the 8-week intervention. Three participants withdrew from the study prior to the 3rd week of the intervention (n=1 for personal reasons, and n=2 due to pain/discomfort due to pre-existing conditions). No participants withdrew from the study due to issues related to the telehealth delivery of the intervention. One participant could not complete the fitness assessments required for the main study but completed the 8 weeks of training and is therefore included in this study. Characteristics for participants who completed the intervention (n=21) are presented in Table 2. The mean (± SD) age was 62.4 y (± 11.2) and 12 (57%) were male. The mean time since stroke was 7.5 y (± 6.8), and all were independent walkers (FAC ≥ 4) with a mean self-selected walking speed of 1.1m/s (± 0.3). The 21 participants who completed the 8-week intervention completed the post-intervention questionnaires (100% response rate). VO_2peak_ improved 12.5% overall (mean 1.9 mL/kg/min, 95% CI 0.7-3.2) from Week 0 to Week 8 (Galloway, 2019).

**Table 2.** Characteristics of Participants who Completed 8 Weeks of Exercise Training (n=21)

Characteristic (n = 21)	
Age *(y)*, mean (SD)	62.4 (11.2)
Gender, number male (%)	12 (57)
Stroke Type, n (%)	
Haemorrhagic	9 (43)
Ischemic	9 (43)
Unknown	3 (14)
Stroke side, number right side (%)	10 (48)
Time since stroke *(y)*, mean (SD)	7.5 (6.8)
Stroke severity mRs,	
Level 0 *n* (%)	2 (10)
Level 1 *n* (%)	1 (5)
Level 2 *n* (%)	9 (43)
Level 3 *n* (%)	8 (38)
Stroke impairment	
FM_LL, mean (SD) (0–34)	27.0 (7.2)
Walking Ability	
Speed, self-sel *(m/s)*, mean (SD)	1.1 (0.3)
Speed, fast *(m/s)*, mean (SD)	1.3 (0.4)
FAC	
Score = 4 *n* (%)	2 (10)
Score = 5 *n* (%)	19 (90)
Living with family, number yes (%)	20 (95)
Psycho-social factors, *mean* (SD)	
Quality of life (EQ-VAS) (0–100)	75.9 (11.1)
Anxiety (HADS) (0 to 21)	4.5 (4.1)
Depression (HADS) (0 to 21)	4.4 (3.1)
Fatigue (FAS) (0 to 50)	21.8 (4.7)
Cognition MoCA, mean (SD) (0–34)	24.9 (3.4)
Physical Activity	
Self-reported, (min/wk) mean (SD)	57.6 (71.8)
Technical Familiarity Score, *mean* (SD) (0–100)	65.9 (24.9)
Score <50, *n* (%)	7 (33)
Score 51–85, *n* (%)	8 (38)
Score >85, *n* (%)	6 (29)

mRs = modified Rankin Score; FM_LL = Fugl-Meyer lower limb; self-sel = self-selected; FAC = Functional Ambulation Classification score; MoCA = Montreal Cognitive Assessment. EQ-VAS = Euroqol 5D-5L health-related quality of life score (higher values better); HADS (lower values better); FAS (lower values better).

### ADHERENCE

There were 504 scheduled exercise sessions; 476 (94%) exercise sessions were completed and 408 (85% of scheduled) sessions were completed via telehealth (by n=20 participants). One participant (technical familiarity score = 51) was supervised in-person for 14 sessions at home after being unable to manage the telehealth connection instructions in the first week of the intervention despite the provision of an additional home visit and telephone support. All other completed sessions were unsupervised (n=54, 11 %), mainly due to participant illness or scheduling difficulties (Table 1).

### ADVERSE EVENTS

One adverse event (a fall requiring medical assistance) occurred during one supervised exercise session. The most common reason for missed sessions was participant illness (Table 1).

### TECHNOLOGY-RELATED OUTCOMES

#### IT AVAILABILITY:

All households of enrolled participants owned a computer, and participants primarily used these for accessing the internet or word processing. Household internet access was via high speed broadband (NBN) for 13 (62%) participants and via lower speed broadband (ADSL) for 8 (38%). Twelve (57%) participants used an iPad to access telehealth during this trial.

#### TECHNICAL FAMILIARITY:

The mean technical familiarity score was 66 (± 24, range 27-98). Six (29%) participants had scores > 85, while seven (33%) scored < 50 (see Table 2). Nine (42%) participants reported being very familiar with computers, however over half reported being either unfamiliar or only slightly familiar. While some participants accessed the internet several times per day, three (14%) participants reported they rarely or never accessed the internet. The internet was used most commonly for browsing websites or sending emails, and 12 (57%) participants used it for both chatting (e.g., Facebook messenger) and video chatting (e.g., Skype).

#### RELIABILITY:

Three hundred seventy-two (78% of scheduled) telehealth sessions were delivered via video-conference, 20 (4%) by phone, and 16 (3%) through a combination of phone and video-conferencing. The latter occurred if internet connections dropped out or became too unstable during a session, video quality was unacceptable, or if an audio delay made communication difficult. Internet drop out or failure, or sub-optimal performance of the internet occurred during 24 (5% of all) sessions (Table 1).

#### QUALITY:

**Figure 1.**
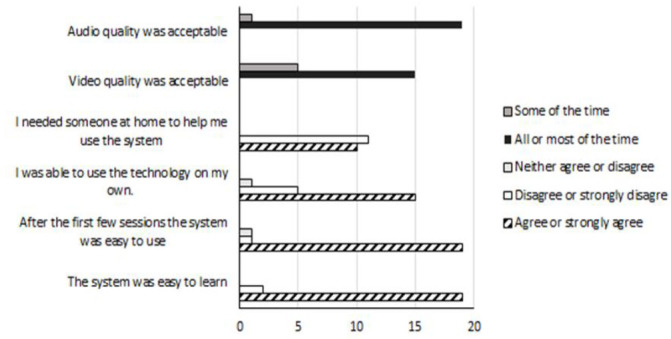
System and quality and usability (participant feedback).

**Figure 2.**
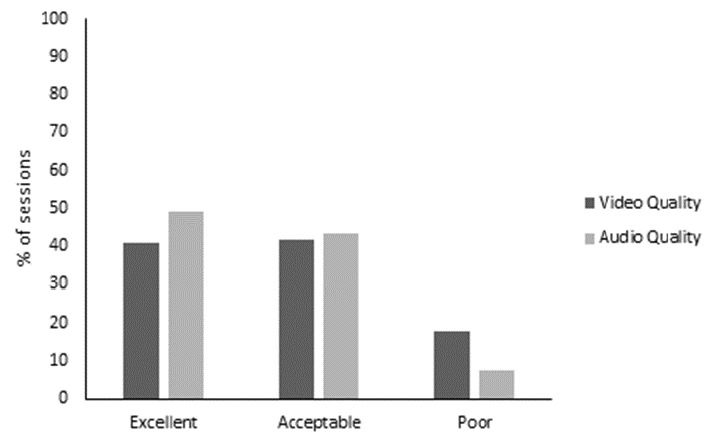
Video and audio quality rated by the exercise instructor.

Audio and video quality were rated as acceptable most or all of the time by 15 (75%) and 19 (95%) participants, respectively (Figure 1) and were rated as acceptable in more than 85% of sessions by the exercise instructor (Figure 2), however the instructor ratings were below excellent for over half the sessions. Instructor ratings of video quality were five times more likely to be excellent when participants had high speed internet access compared with lower speed broadband. Similarly, audio quality ratings were twice as likely to be excellent with high speed internet, compared with lower speed broadband.

#### USABILITY:

Most participants agreed or strongly agreed that the telehealth system was easy to learn (n=19, 95%) and easy to use after the first few sessions (n=19, 95%). While half the participants reported they needed someone at home to help, most (n=15, 75%) agreed that they were actually able to use the system by themselves (Figure 1). Of the six participants who reported not being able to use the system by themselves, four scored below 50 on the technical familiarity scale, and the remaining two had low Fugl-Meyer lower limb (FM_LL) scores, indicating a higher level of physical impairment.

### SATISFACTION OUTCOMES

#### SATISFACTION WITH TELEHEALTH DELIVERY:

**Figure 3.**
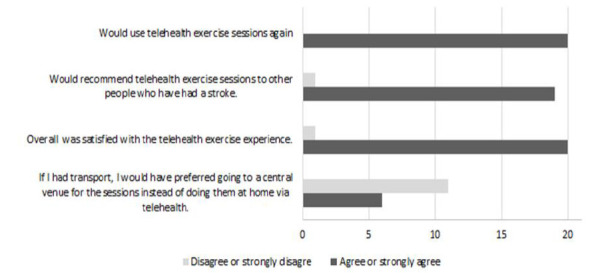
Participant satisfaction with telehealth

All participants who completed the eight weeks of telehealth-supervised exercise (n=20) would use telehealth exercise sessions again and were satisfied overall with the experience. All participants agreed or strongly agreed they felt safe during exercise sessions. Most (95%) would recommend telehealth exercise sessions to other people who have had a stroke (Figure 3). All participants reported they had sufficient space at home to do the planned exercises, could see the instructor at the same time, and felt safe during exercise sessions. Over half the participants preferred exercising at home even if transport had been available, and most disagreed they would have preferred to do some of the sessions without telehealth supervision.

Participants were also asked to comment on what (if anything) they liked about telehealth. Comments were categorised according to the following themes: participant benefits, convenience, instructor-related, altruism, and satisfaction. Thirty-eight percent of comments were in relation to the benefits participants perceived for themselves, such as providing the discipline and motivation to exercise, self-confidence, improved fitness, health and function, and computer skills. Convenience also rated highly (20% of comments). Comments included “it was convenient to have a day at home but still (be) supervised” and “not needing to travel.” Twenty-one percent of comments were specifically about the instructor, with participants liking that the instructor provided motivation and expertise. Comments on satisfaction (18%) included “I enjoyed the sessions very much”, and “there was excitement to wake up and do it” (i.e., the telehealth exercise session). Participants were also asked if there was anything they disliked. Most (81%) reported that there was nothing they didn't like about telehealth. The remainder disliked some aspects of the technology, particularly the heart rate monitors. Both types were problematic, with comments such as “heart rate monitor easy to use but slow to get a reading (pulse oximeter),” and in regards to the Garmin monitor, participants commented on the difficulty of managing the device, such as “too many buttons to press, difficult with left hand.” Most other negative comments were in regard to internet speed and reliability. Technical familiarity, age, or disability level did not affect participant satisfaction.

#### SATISFACTION WITH CONTENT AND DOSE OF THE EXERCISE PROGRAM:

While all participants agreed or strongly agreed the exercises were challenging enough to improve their fitness, the program had enough variety, and they had enough equipment at home to do the prescribed exercises, six (28%) participants found the exercises difficult to perform due to their physical ability (e.g., limb weakness). The preferred dose parameters for the telehealth exercise program were: frequency 3x/week (preferred by 76% of participants); session duration 20-30 min (67%); and program length 6-8 weeks (34%) or 8-12 weeks (34%); 19% would have preferred more than 12 weeks. Some participants (29%) would have preferred to go to a central venue rather than exercise at home via telehealth if they had transport.

A complete summary of the responses to the two questionnaires are included in the Supplementary Material.

## DISCUSSION

We found that the telehealth delivery of supervised exercise was feasible in terms of retention, adherence, safety, reliability, and usability for our sample of ambulant community-dwelling stroke survivors, from both the perspective of the participants and the research team, and was acceptable to the participants. Telehealth delivery did, however, impact on recruitment for the exercise trial.

Prior familiarity with technology, age, or level of disability were not barriers to successfully participating in telehealth supervised exercise sessions. Exercising with supervision provided via telehealth was safe, and adherence to the exercise program was high. The vast majority of exercise sessions in this trial were delivered successfully using video-conferencing, and participants found the systems easy to learn and use. Participants were satisfied overall with the telehealth experience and would recommend telehealth exercise sessions to others who have had a stroke. Participants also derived many personal benefits by participating, and liked the convenience telehealth provided. If given a choice, most would prefer to have telehealth sessions available 3 d/week for 20-30 min, however there was no consensus on the ideal program length.

The safety of participants during the exercise trial was paramount given the range of ability levels and comorbidities people present with after stroke. Hemiplegia, reduced balance, cognitive impairment and a high incidence of cardiovascular risk factors are all common and we deemed it unsafe and unethical to allow participants to be supervised via telehealth without another adult present at home during sessions in case of emergency. We did find that many people who expressed an interest in participating in this form of exercise lived alone or did not have family members or carers available or able to assist, and this is a potential barrier for future participation in home-based telehealth-supervised exercise in this population. Provided adequate consideration is given to factors such as medical suitability (screening), emergency procedures, risk stratification, and risk assessments of the home, telehealth exercise sessions for this population could potentially be conducted safely without the requirement that another adult be present during sessions.

For those who had a home environment deemed safe for the current trial, telehealth exercise sessions were feasible and adherence was high. Some participants felt that telehealth made them accountable and provided the discipline they needed to exercise regularly, and this almost certainly contributed to the high adherence amongst participants, as was found in trials in other clinical populations (Hwang et al., 2015).

Although feasible overall, there were some factors that detracted from the experience for some participants. More than a third of participants in this trial had lower speed broadband at their location, and this affected video and audio quality. While video and audio quality did not influence participants' overall level of satisfaction with the program, normal conversation was affected during these sessions, and this may be a potential barrier for people with aphasia post-stroke. The range of technical familiarity amongst participants was broad, and the majority of participants who required someone at home to help them use the telehealth system had low levels of technical familiarity. Encouragingly, despite a third of participants scoring less than 50% on the technical familiarity scale, most found the system easy to learn and were able to operate it on their own after the initial few sessions. This is an important finding given the increased interest in telerehabilitation, and the preconception of many older people that they may find access to telehealth services difficult due to their age, technical familiarity or level of impairment (Crotty et al., 2014; Shulver, Killington, Morris, & Crotty, 2017). The findings from this trial provide evidence that there were few barriers (such as prior level of technical familiarity, stroke-related impairments, family support, or confidence) to the successful delivery and uptake of telehealth-supervised exercise identified in this population.

Overall, the level of satisfaction and enjoyment expressed by participants was high. Participants perceived both physical (improved fitness, function, and sleep) and psychological benefits (improved self-confidence, motivation, discipline to exercise, and goal achievement) and therefore quality of life post-stroke may be higher for these people. Many commented favourably on the convenience telehealth provided. It decreased the burden of transport, often cited as the major barrier for stroke survivors and other clinical populations to access centre-based exercise programs (Marzolini et al., 2016; Nicholson et al., 2013), and many participants liked that telehealth sessions were quick and left the rest of their day free.

We found that delivering exercise sessions aimed at improving fitness to people after stroke via telehealth was highly feasible for those who participated, and contributed to lowering secondary stroke risk in most participants. While we are unaware of any similar trials published in stroke (Chen et al., 2015; Sarfo et al., 2018) our results are in agreement with findings from other clinical populations. A systematic review of telehealth fitness programs in people with chronic heart failure found that the prevalence of adverse events was similar to centre-based programs (Hwang et al., 2015), and in cardiac rehabilitation trials, telehealth programs were at least as effective as usual care in improving cardiovascular risk factors and functional capacity (Chan, Yamabayashi, Syed, Kirkham, & Camp, 2016; Clark et al., 2015; Rawstorn, Gant, Direito, Beckmann, & Maddison, 2016; Southard et al., 2003). In regards to age and level of technical familiarity our finding are also in agreement with other trials (Shulver et al., 2017) and highlight that most participants coped well with the technology, regardless of their prior level of technical familiarity.

Participant preference for session frequency and duration were similar to the intervention parameters, however almost a third would have preferred in-person supervision to telehealth if transport was not an issue. This suggests that telehealth should not be the only delivery method explored to overcome the transport barrier post-stroke, and consideration should also be given to other ways to overcome this barrier, such as providing transport to other community exercise programs.

## LIMITATIONS OF STUDY

We included only ambulant people with mild-moderate impairments who were, on average, many years post-stroke, and therefore the results may not be generalisable to more acute stroke survivors, or to those with higher levels of physical or cognitive impairment. We also included only people who had volunteered to participate based on their belief they had the required ability and skill level to participate in telehealth. The sample size was small, and the questionnaires used were only assessed for face validity. The study was conducted in one geographical region of Australia, and the feasibility of providing a similar intervention may be affected by location and available infrastructure elsewhere. No comparison was made to other delivery methods, and only one instructor provided all the telehealth sessions.

## CLINICAL IMPLICATIONS

Telehealth delivery of exercise sessions to people after stroke appears feasible, and may provide an acceptable alternative for providing supervised exercise to people after stroke. While telehealth did restrict recruitment to this exercise trial, once recruited, neither age nor prior familiarity with technology affected participants' ability to participate, and are not likely deterrents for future use. Telehealth eliminated the need for transport, and could make accessing supervised exercise more achievable for rural dwelling stroke survivors and for others who have difficulty accessing community fitness facilities. As access to higher speed internet increases over time, the user experience is likely to improve further. The findings from this study can be used to inform the development of future telehealth trials of exercise for people after stroke.

## Figures and Tables

**Supplementary Table 1.** Technical Familiarity Questionnaire - Summary of responses (n=21)

	**Response**	**Number**	**%**
**A: Computer**			
Does your household own a computer	Yes	21	100
How familiar are you with using a computer	Very familiar	9	43
	Slightly familiar	7	33
	Quite familiar	1	5
	Unfamiliar	4	19
Do you use a computer for any of the following tasks	Word processing	13	62
	Using the internet	19	91
	Carrying out work-related tasks	11	52
	Carrying out home related tasks	9	43
	Reading about my symptoms or illnesses	13	62
How easily can you acquaint yourself with a new computer program	I find it easy	9	45
	I have some difficulty	5	25
	I have major difficulty	3	15
	I find it impossible/I don't make use of new programs	3	15
	No response	1	1
Since your stroke have you ever installed a computer program on your own?	Yes	9	43
	No	12	57
Since your stroke have you ever used a tablet (e.g. iPad)	Yes	16	76
	No	5	23
**B:Internet**			
What type (s) of internet access do you have at home?	Broadband (e.g. ADSL2)	8	38
	NBN (high speed broadband)	13	62
	Low speed (e.g. via modem or ISDN)	1	5
	Via mobile phone	2	10
	Via wireless dongle	0	0
	I have no internet access	0	0
	I have internet access but I don't know the type of access	0	0
How often do you use the internet?	Several times a day	12	57
	Once a day	2	9
	Several times a week	3	14
	Once a week	1	5
	Rarely/never	3	14
Internet Tasks	Browsing websites	17	85
	Sending email	16	79
	Chatting (e.g. using Facebook messenger)	12	57
	Video chatting (e.g. Skype)	12	57
	Getting information about my symptoms/illnesses	11	52
	Communicating with doctors	1	5
	Communicating with others with stroke	4	19
How often do you send emails?	Several times a day	5	24
	Once a day	0	0
	Several time a week	7	33
	Once a week	2	10
	Rarely/never	7	33
How often do you read emails?	Several times a day	8	38
	Once a day	7	33
	Several time a week	0	0
	Once a week	1	5
	Rarely/never	5	24
**C: Mobile phone**			
Do you own a mobile phone?	Yes	18	86
	No	3	14
Do you own a Smart Phone (one that can browse the internet, send emails etc.)?	Yes	12	57
	No	9	43
How often do you use a mobile phone?	Several times a day	14	67
	Once a day	0	0
	Several time a week	0	0
	Once a week	3	14
	Rarely/never	4	19
Mobile Phone Tasks	Calling	19	91
	Text messaging	13	62
	Audio or video messaging	6	29
	Browsing websites	7	33
	Reading or sending emails	4	19
	Planning or scheduling	8	38
	Social Media (e.g., Facebook, Twitter or other )	8	38

**Supplementary Table 2.** Telehealth Usability and Satisfaction Questionnaire- Summary of responses (n=21)

Item		Number	%
**Quality**			
Which system did you use?	EHab App (iPad)	10	48
	Neorehab website	8	38
	Both	1	5
	Unsure	2	10
	Other (specify	0	0
The video quality was acceptable.	All of the time	7	33
	Most of the time	8	38
	Some of the time	5	24
	Rarely	0	0
	Never	0	0
	N/A	1	5
The audio quality was acceptable.	All of the time	9	43
	Most of the time	10	48
	Some of the time	1	5
	Rarely	0	0
	Never	0	0
	N/A	1	5
**Usability**			
The system was easy to learn.	Strongly agree	14	67
	Agree	5	24
	Neither agree or disagree	0	0
	Disagree	1	5
	Strongly disagree	1	5
After the first few sessions the system was easy to use	Strongly agree	15	71
	Agree	4	19
	Neither agree or disagree	1	5
	Disagree	1	5
	Strongly disagree	0	0
I was able to use the technology on my own.	Strongly agree	8	38
	Agree	7	33
	Neither agree or disagree	1	5
	Disagree	4	19
	Strongly disagree	1	5
I needed someone at home to help me use the system.	Strongly agree	6	28
	Agree	4	19
	Neither agree or disagree	0	0
	Disagree	6	29
	Strongly disagree	5	24
**Exercise Program**			
I found the exercises difficult to perform due to my physical ability (e.g., limb weakness).	Strongly agree	1	5
	Agree	5	24
	Neither agree or disagree	1	5
	Disagree	7	33
	Strongly disagree	7	33
The exercises were challenging enough to improve my fitness.	Strongly agree	10	48
	Agree	11	52
	Neither agree or disagree	0	0
	Disagree	0	0
	Strongly disagree	0	0
The exercise program had enough variety.	Strongly agree	13	62
	Agree	7	33
	Neither agree or disagree	1	5
	Disagree	0	0
	Strongly disagree	0	0
I felt safe doing the exercises	Strongly agree	14	67
	Agree	7	33
	Neither agree or disagree	0	0
	Disagree	0	0
	Strongly disagree	0	0
I had enough equipment at home to do the exercises.	Strongly agree	13	62
	Agree	8	38
	Neither agree or disagree	0	0
	Disagree	0	0
	Strongly disagree	0	0
I had enough space at home to both do the exercises and see the instructor at the same time.	Strongly agree	15	71
	Agree	6	29
	Neither agree or disagree	0	0
	Disagree	0	0
	Strongly disagree	0	0
I would have preferred to do some of the exercise sessions by myself without being supervised by telehealth.	Strongly agree	0	0
	Agree	2	10
	Neither agree or disagree	3	14
	Disagree	7	33
	Strongly disagree	9	43
Which heart rate monitor (s) did you use?	Chest strap and watch (Garmin)	2	10
	Finger clip (Pulse oximeter)	14	67
	Both	5	24
	Neither	0	0
The heartrate monitor I used the most was easy to use.	Strongly agree	11	53
	Agree	7	33
	Neither agree or disagree	0	0
	Disagree	1	5
	Strongly disagree	2	10
**Exercise preference**			
**If you had a choice:**			
What is your preferred length of time for a telehealth supervised exercise session?	Less than 15 minutes	0	0
	15–20 minutes	2	10
	20–30 minutes	14	67
	30–45 minutes	4	19
	More than 45 minutes	1	5
What is your preferred number of telehealth supervised exercise sessions per week?	None	0	0
	1	0	0
	2	2	10
	3	16	76
	4	2	10
	5 or more	1	5
What is your preferred length of time for a telehealth supervised exercise program?	Less than 4 weeks	0	0
	4–6 weeks	3	14
	6–8 weeks	7	33
	8–12 weeks	7	33
	More than 12 weeks	4	19
**Satisfaction**			
If I had transport, I would have preferred going to a central venue for the sessions (e.g., physio clinic, community centre, gym) instead of doing them at home via telehealth.	Strongly agree	0	0
	Agree	6	29
	Neither agree or disagree	4	19
	Disagree	4	19
	Strongly disagree	7	33
Overall I was satisfied with the telehealth exercise experience.	Strongly agree	15	71
	Agree	5	24
	Neither agree or disagree	1	5
	Disagree	0	0
	Strongly disagree	0	0
I would recommend telehealth exercise sessions to other people who have had a stroke.	Strongly agree	15	75
	Agree	4	20
	Neither agree or disagree	0	0
	Disagree	1	5
	Strongly disagree	0	0
I would use telehealth exercise sessions again	Strongly agree	16	75
	Agree	4	19
	Neither agree or disagree	0	0
	Disagree	0	0
	Strongly disagree	1	5
